# Utilization of recombinase polymerase amplification combined with a lateral flow strip for detection of *Perkinsus beihaiensis* in the oyster *Crassostrea hongkongensis*

**DOI:** 10.1186/s13071-019-3624-3

**Published:** 2019-07-24

**Authors:** Lin Wu, Lingtong Ye, Zhaorui Wang, Yingyi Cui, Jiangyong Wang

**Affiliations:** 1 Key Laboratory of Aquatic Product Processing; Key Laboratory of South China Sea Fishery Resources Exploitation & Utilization, Ministry of Agriculture and Rural Affairs; South China Sea Fisheries Research Institute, Chinese Academy of Fishery Sciences, Guangzhou, 510300 China; 2 Shanghai Ocean University, Shanghai, 201306 China; 3 College of Fisheries, Tianjin Agricultural University, Tianjin, 300384 China; 4 Zhongshan Center for Animal Disease Prevention and Control, Zhongshan, 528455 Guangdong China

**Keywords:** Recombinase polymerase amplification, Lateral flow strips, Rapid detection method, *Perkinsus beihaiensis*, *Crassostrea hongkongensis*

## Abstract

**Background:**

Perkinsosis, a disease caused by the protist *Perkinsus*, is responsible for mass mortalities of many molluscan species worldwide. The rapid, early and accurate detection of *Perkinsus* infection is necessary to react to outbreaks, and manage disease transmission. Current methods for diagnosis of *Perkinsus* spp. are time-consuming or require professional equipment and experienced personnel, rendering them unsuitable for field application. Recombinase polymerase amplification (RPA) assay is a highly sensitive and selective isothermal amplification technique that operates at temperatures of 37–42 °C, requires minimal sample preparation, and is capable of amplifying as low as 1–10 target DNA copies in less than 20 minutes.

**Methods:**

We report a novel RPA assay that amplifies the internal transcriber spacer (ITS) region of *P. beihaiensis*, which, followed by rapid detection of amplicons using a lateral flow (LF) strip, enables easy visualization of results by the naked eye.

**Results:**

The LF-RPA assay successfully amplified *P. beihaiensis* DNA using a set of primers of 20–25 bp in length. After incubation at 37 °C for 25 min, results were read within 5 min by the naked eye on a lateral flow strip. Our LF-RPA assay was comparably sensitive to qPCR assay, and capable of detecting as few as 26 copies of *P. beihaiensis* DNA. Cross-amplification occurred with other two *Perkinsus* species, *P. olseni* and *P. chesapeaki*, but not with other potential pathogen taxa in culture environments. We compared the performance of LF-RPA, conventional PCR and qPCR assays on 60 oyster samples. While LF-RPA assay results were 86.2% as sensitive, 77.4% as specific, and generally in agreement with those of conventional PCR results, they were more (93.3%) sensitive, (86.7%) specific, and agreed better with qPCR assay results. Future research should focus on developing simple DNA extraction methods that do not require professional laboratories and complicated extraction procedures, to facilitate application of this LF-RPA assay in the field.

**Conclusions:**

Our LF-RPA assay provides a rapid and efficient method for detecting species of *Perkinsus*. This novel assay has potential to be used in field applications.

## Background

Perkinsosis, a disease caused by the protist *Perkinsus*, has caused mass mortalities in species of mollusc worldwide [1, 2]. Most *Perkinsus* species are generalist parasites, have low host specificity, can switch between hosts and are geographically widespread [3, 4]. For example, *P. olseni*, an internationally reportable or notifiable molluscan pathogen [World Organization for Animal Health (OIE)], is known from 17 countries and 27 host species in five orders and six families [5].

The life-cycles of *Perkinsus* spp. involve direct transmission between the molluscan host without an intermediate host. All life stages (trophozoite, hypnospore and zoospore) are infective [6]. Without surveillance, widespread transmission of *Perkinsus* spp. can occur anywhere molluscs (especially oyster species, e.g. *Crassostrea gigas*, *C. virginica* and *C. hongkongensis*) are cultivated, particularly through movements of infected hosts during aquaculture production, and in trade. Given the economic loss associated with tissue degradation and potential lethality of *Perkinsus* spp. to host species, and the difficulties of applying chemotherapeutants in open-sea culture environments, the rapid, early and accurate detection of *Perkinsus* spp. is essential to control its transmission and manage outbreaks.

The current gold standard for detection and quantification of *Perkinsus* spp. in marine molluscs involves incubation of host tissues in Ray’s fluid thioglycollate medium (RFTM) [7]. Fresh molluscan tissue is added to a tube containing RFTM (for medium components and concentrations see [7]) and incubated in darkness at room temperature (20–25 °C) for 4–7 days; after incubation, the tissue is digested with 2-M NaOH, and washed several times with phosphate-buffered saline (pH 7.2); the resultant tissue is used to detect and quantify *Perkinsus* spp. by counting hypnospores using a haemocytometer beneath a microscope. This technique lacks specificity, is time-consuming and must be performed using fresh tissue [8–10]; it is also only sensitive to *P. marinus* at concentrations exceeding 1000 cells per gram of wet oyster tissue [10, 11]. Other diagnostic methods for detection of *Perkinsus* spp. include: immunoassays using monoclonal and polyclonal antibodies [12], histopathological assays [13], real-time polymerase chain reaction assay (qPCR) [14, 15], loop-mediated isothermal amplification (LAMP) assay [10] and *in situ* hybridization assay (ISH) [16]. Most of these assays are time consuming, and must be performed in laboratories, requiring costly infrastructure, reliable electrical supplies and skilled staff. Of these, LAMP, an isothermal amplification method that uses enzymes instead of thermal cycling for DNA synthesis, has been recently applied in detection of *Perkinsus* spp. infection in molluscs. This assay must be performed at temperatures exceeding room temperature (60–65 °C), requires complicated primers and completion of the detection process takes at least 49 minutes [10].

Recombinase polymerase amplification (RPA) is a highly sensitive and selective isothermal amplification technique that can be performed between 37 and 42 °C, requires minimal sample preparation and is capable of amplifying as few as 1–10 target DNA copies within 20 minutes [17, 18]. RPA can be detected by agarose gel electrophoresis (AGE), or in real-time using TwistAmp TM exo probes (TwistDx, Cambridge, UK) [19, 20]. Alternatively, oligochromatographic lateral flow (LF) strips can be used, the recognition of which is achieved by binding to a tag-specific antibody on the surface at the detection line and cross-linking it with a second tag-specific antibody on colloid gold nanoparticles, resulting in a coloured signal that can be semiquantitatively analysed and visualised by the naked eye [21]. As the LF strip assay takes 5–10 minutes to complete, requires no special equipment and can be visualised by the naked eye, combining it with RPA would create a sensitive, specific and quick system to identify the existence of *Perkinsus* in the field [22].

Given the advantages of RPA, this assay has been used to diagnose various pathogens, such as viruses [23–25], bacteria [19, 26, 27] and parasites [21, 22, 28]. However, most RPA assays have focused on detection of pathogens related to humans or domestic animals, and not aquatic animals [20, 29]. We are unaware of any published reports using this assay in which *Perkinsus* has been detected in molluscs. Accordingly, we developed a combined isothermal RPA and lateral flow strip detection assay (LF-RPA) for the detection of *P. beihaiensis* in the oyster *C. hongkongensi.* We evaluated the sensitivity of this LF-RPA assay by comparing it with that of qPCR and conventional PCR methods using McNemar’s Chi-square tests. Degrees of agreement among LF-RPA, qPCR and conventional PCR test results were measured using kappa (K) values.

## Methods

### Sample collection and DNA extraction

A total of 150 oysters (*C. hongkongensis*) were collected from farms in Zhanjiang, Guangdong Province, China, where *P. beihaiensis* was prevalent in local molluscs (e.g. *C. hongkongensis*, *C. ariakensis*, *Soletellina acuta*) [15, 30]. Oyster gills were excised using forceps and fixed in 95% (v/v) ethanol. Approximately 200 mg samples of gill tissue were cut and placed in 1.5-ml Eppendorf tubes. After evaporation of ethanol, samples were subjected to DNA extraction procedures using a HiPure Mollusc mini DNA Kit (Magen, Guangzhou, China) following the manufacturer protocols. DNA quality and concentration were measured using a NanoDrop 2000 spectrophotometer (Thermo Fisher Scientific, Waltham, MA, USA); extracted DNA was stored at − 20 °C.

### Conventional PCR detection

Diagnosis of *Perkinsus* spp. infection in oyster samples was performed using conventional polymerase chain reaction (PCR) amplification of the internal transcriber spacer (ITS) region, with *Perkinsus* genus-specific primer pairs PerkITS-85 and PerkITS-750 (Table 1) [31]. PCR was performed in a 25 μl reaction comprising 12.5 μl of PCR Mix (Dongsheng Biotech, Guangzhou, China), 1 μl of each primer (10 μM), 1 μl of extracted DNA and 9.5 μl of ultrapure water. PCR results were confirmed on a 1% agarose gel stained with SYBR green. A positive control using 1000 copies of *P. beihaiensis*-pMD 18T recombinant plasmid DNA and a negative control (nuclease-free water) were included in each run. Positive PCR products were sequenced to determine the specific *Perkinsus* species. When the target sequence was 99.5–100% identical to *P. beihaiensis* (GenBank accession no. JN054741), we considered it positive for infection of *P. beihaiensis* in the sample.

**Table 1:** Primers and probe for the conventional PCR, qPCR and LF-RPA

Assay	Primer/probe name	Sequence (5′–3′)	Product size (bp)	References
PCR	PerkITS-85	CCGCTTTGTTTGGATCCC	703	[31]
	PerkITS-750	ACATCAGGCCTTCTAATGATG		[31]
qPCR	Q2-F	TCGATGAAGGACGCAACGAA	291	Present study
	Q2-R	CTCATTTCTGCGGGGCTACA		
LF-RPA	Pits6-F	CGATGAAGGACGCAACGAAGTG	186	Present study
	Pits6-R	biotin-CAAGCGGGATACAAAGCATTAGATT		
	Probe	FAM-CAGAATTCCGTGAACCAGTAGAAATCTCAA CGCA-(THF)-TACTGCACAAAGGGGA-/C3-spacer/		

*Abbreviations*: LF, lateral flow; RPA, recombinase polymerase amplification

A total of 60 oyster samples (29 *P. beihaiensis* positive and 31 *Perkinsus* spp. negative) were used to evaluate the diagnostic performance of the RPA assay. These samples were also detected by a real time PCR assay (qPCR).

### qPCR detection

The primers Q2-F and Q2-R (Table 1) were designed for the qPCR assay, which amplified a 291 bp ITS fragment of *P. beihaiensis*. qPCR sensitivity was determined using 10-fold serial dilutions of *P. beihaiensis*-pMD18T recombinant plasmid DNA (2.6 × 10^7^ to 2.6 × 10^1^ copies/μl). The qPCR was carried out in a 10 μl reaction, comprising 5 μl TB Green™ Premix (TaKaRa, Kusatsu, Japan), 0.5 μl of each primer (10 μM), 1 μl of extracted DNA and 3 μl of nuclease-free water. Reactions were conducted in triplicate on an Eco™ Real-Time PCR System (Illumina, San Diego, CA, USA) using the Eco System Software with the following temperature program: 95 °C for 30 s; 40 cycles of 95 °C for 5 s and 60 °C for 40 s. In all reactions *P. beihaiensis* DNA was used as a positive control, and DNase-free water as the non-template control. A sample was considered positive if the qPCR threshold cycle (CT) value of the tissue rose above zero before the 35th cycle, and negative if the CT value was zero before the 35th cycle.

### RPA detection

#### Primer and probe design

The highly repeated ITS region of *P. beihaiensis* (GenBank accession no. JN054741) was selected as a target sequence. Candidate RPA primers were first sought using Primer Premier v.5.0 software (Premier Biosoft, Palo Alto, CA, USA) following TwistAmp® reaction kit (TwistDx) guidelines (the best primers are 30–35 bases in length and have 30–70% GC content). No suitable RPA primers were screened, as gel-electrophoresis results lacked bands or produced unequally sized bands for the target fragment. Of 10 pairs of candidate RPA primers, one pair of efficient RPA primers (Pits6-F and Pits6-R, amplicon size 186 bp) was subsequently screened using primers of normal length (20–25 bases) (Table 1). For adaptation to the lateral flow detection system, the 5′ end of the reverse primer (Pits6-R) was labelled with a biotin (Table 1). Of 5 candidate RPA probes, one probe was screened out and its 5′ end labelled with a carboxyfluorescein (FAM) group, a C3 spacer (SpC3) on the 3′ end, and a tetrahydrofuran (THF) residue to replace an internal base (Table 1).

#### RPA assays

A TwistAmp Basic kit (TwistDx) was used to screen the best combinations of RPA primers following the manufacturer’s instructions. Each 50 μl reaction contained 29.5 μl of rehydration buffer, 2.4 μl of each RPA forward and reverse primer (10 μM), 12.2 μl of dH_2_O and 1 μl of DNA template; 2.5 μl of magnesium acetate (280 mM) was added to initiate the reaction. A positive control using 1000 copies of *P. beihaiensis*-pMD 18T recombinant plasmid DNA and a negative control (nuclease-free water) were included in each run. Reaction tubes were briefly centrifuged and vortexed to mix reagents, then incubated for 25 min in a heat block at 37 °C. After incubation, reaction products were purified using VAHTSTM DNA clean beads (Nanjing Vazyme Biotech Co., Nanjing, China) and visualised on a 2.5% agarose gel. Candidate RPA primers were regarded as suitable if purified RPA products were evident, a single band was apparent, the band size was the same as that of the primer-designed target fragment, and negative and non-template controls lacked bands.

#### Lateral-flow strip RPA assay (LF-RPA)

A TwistAmp nfo kit (TwistDx) was used in combination with HybriDetect 1 strips (Milenia Biotec, Giessen, Germany) to detect *P. beihaiensis* infection in oyster samples. The reaction procedure was similar to that for the TwistAmp Basic kit. To avoid contamination, all reaction procedures were performed in separate biological safety cabinets or pipetting hoods. Each 50 μl reaction contained 29.5 μl of rehydration buffer, 2.1 μl of RPA forward primer (10 μM), 2.1 μl of RPA biotinylated reverse primer (10 μM), 0.6 μl of FAM-labelled probe (10 μM), 12.2 μl of dH_2_O and 1 μl of DNA template; 2.5 μl of magnesium acetate (280 mM) was added to initiate the reaction. In all reactions 1000 copies of *P. beihaiensis*-pMD 18T recombinant plasmid DNA was used as a positive control, and DNase-free water as the non-template control. The reaction tube was incubated for 25 min in a heat block at 37 °C. After incubation, 10 μl of reaction product was diluted in 100 μl of running buffer to test the HybriDetect 1 lateral-flow strips. The strip was placed vertically into the diluted solution, then incubated at room temperature; the final result was read at 5 min. A result was regarded positive when the control and test lines were both visible and negative when only the control line was visible.

### Evaluation of specificity and sensitivity

To determine primer specificity, DNA of seven common pathogens [*P. beihaiensis*, *P. olseni*, *P. chesapeaki*, *Vibrio harveyi*, *Streptococcus agalactiae*, ostreid herpesvirus 1(OsHV-1) and abalone herpes-like virus (AbHV)] of aquatic animals were used as templates. Pathogens were provided by the Fishery Organism Disease Control Division, South China Sea Fisheries Research Institute, Chinese Academy of Fishery Sciences. Each of *P. beihaiensis*, *P. olseni* and *P. chesapeaki* were obtained from molluscs in China’s coastal waters during a *Perkinsus* infection surveillance program performed by the authors (unpublished data); *V. harveyi* was provided by Dr Ruixuan Wang, *S. agalactiae* by Dr Youlu Su, OsHV-1 and AbHV by Dr Jingzhe Jiang.

The sensitivity of RPA, LF-RPA and qPCR assays was assessed using serial dilutions of *P. beihaiensis*-pMD18T recombinant plasmid DNA. Recombinant plasmids (including *P. beihaiensis*-, *P. olseni*- and *P. chesapeaki*-pMD18T) were constructed according to the method provided by Cui et al. [15]. qPCR standard curves were obtained in which CT values of each sample were plotted against the logarithm of the DNA starting concentration. The detection limit was determined as the lowest concentration (within the linear range) that produced an amplification signal on the Eco™ Real-Time PCR System.

### Data analysis

Experimental data were analysed using SPSS Statistics v.19 (IBM Corporation, Armonk, NY, USA). Specificity, sensitivity and accuracy of the LF-RPA assay performance were evaluated. McNemar’s Chi-square test was used to compare sensitivities of LF-RPA, qPCR and conventional PCR assays. Degrees of agreement among LF-RPA, qPCR and conventional PCR test results were measured using kappa (*K*) values, with *K* < 0.4 considered to be a poor agreement and *K* ≥ 0.75 considered to be a good agreement. Statistical significance was set at *P *< 0.05.

## Results

### Specificity

**Fig. 1:**
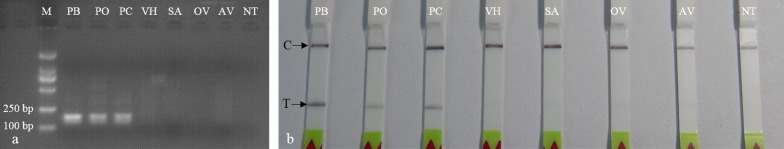
Specificity of RPA and LF-RPA assays. Agarose gel electrophoresis of RPA purified products (**a**) and lateral-flow strip (**b**), showing successful amplification or visual detection of *Perkinsus beihaiensis*, *P. olseni* and *P. chesapeaki*, but not other potential pathogen DNAs. *Abbreviations*: AV, abalone herpes-like virus; C, control; LF, lateral flow; M, marker DL2000; NC, negative control (distilled water); OV, ostreid herpesvirus 1; PB, *P. beihaiensis*; PC, *P. chesapeaki*; PO, *P. olseni*; SA, *Streptococcus agalactiae*; RPA, recombinase polymerase amplification; T, test line; VH, *Vibrio harveyi*

The specificity of RPA and LF-RPA primers was evaluated using DNA of three recombinant plasmids (*P. beihaiensis*, *P. olseni* and *P. chesapeaki*) and four potential pathogens of aquatic animals (*V. harveyi*, *S. agalactiae*, OsHV-1 and AbHV). Both RPA and LF-RPA assays discriminated DNAs of three *Perkinsus* species from those of other pathogens (Fig. 1). RPA and LF-RPA assays designed for *P. beihaiensis* also detected *P. olseni* and *P. chesapeaki*.

### Sensitivity

**Fig. 2:**
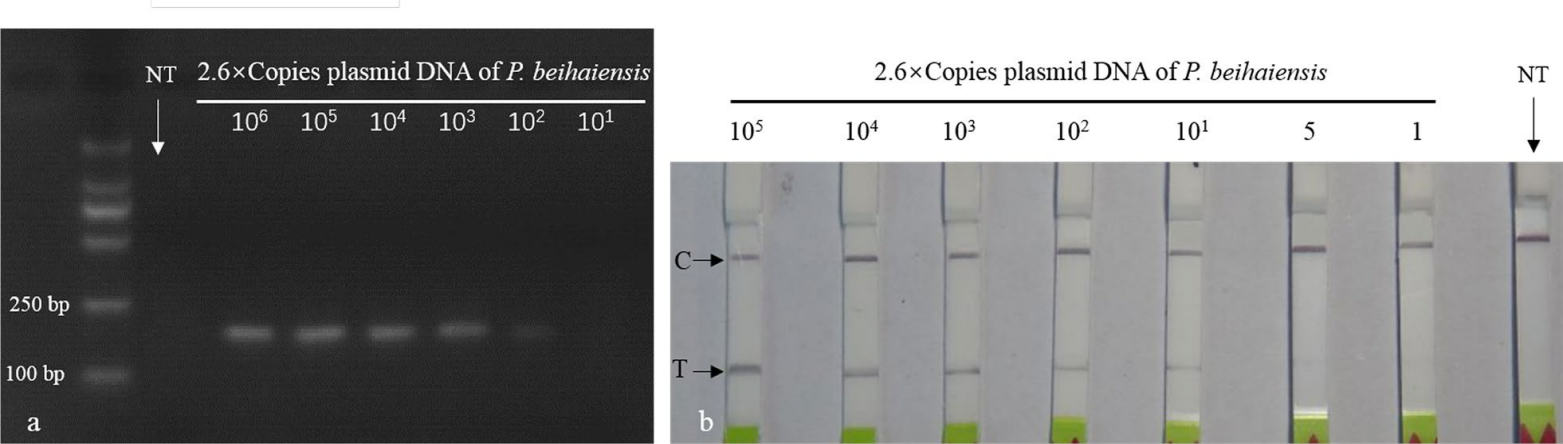
Sensitivity of RPA and LF-RPA assays for detecting *Perkinsus beihaiensis*. **a** RPA products, detection limit 260 copies of plasmid DNA on a stained agarose gel (2.5%). **b** LF-RPA assay, detection limit 26 copies of plasmid DNA on the lateral-flow strip. *Abbreviations*: C, control; LF, lateral flow; NC, negative control (distilled water); RPA, recombinase polymerase amplification; T, test line

**Fig. 3:**
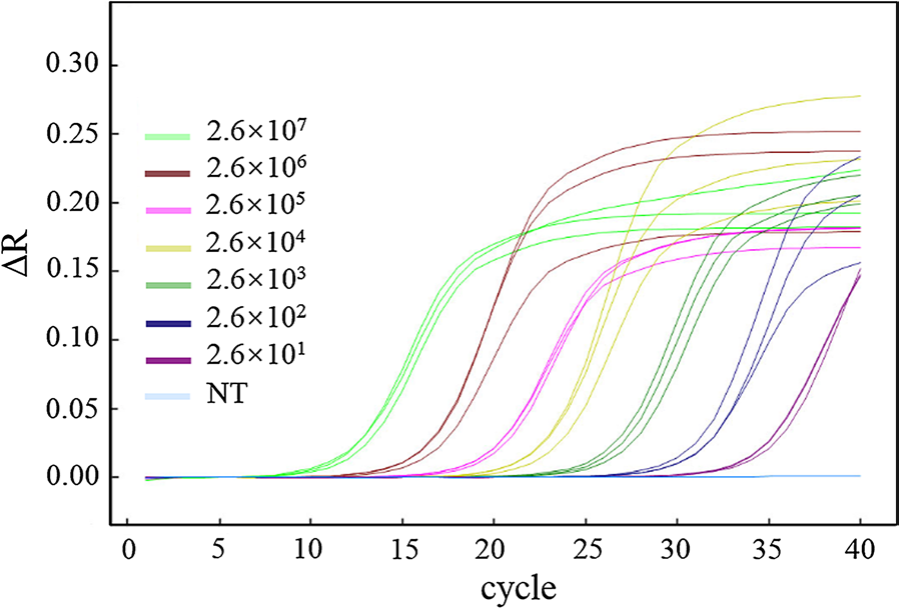
Sensitivity of qPCR. 10-fold serial dilutions of *Perkinsus beihaiensis* plasmid DNA used to determine detection limit (26 copies of *P. beihaiensis* plasmid DNA). *Abbreviations*: NC, negative control (distilled water)

The RPA assay was least sensitive, with a lower detection limit of 260 copies of *P. beihaiensis* plasmid DNA (Fig. 2a). The LF-RPA assay had a detection limit as low as 26 copies (Fig. 2b), comparable to that of the universally recognised, highly sensitive qPCR assay (Fig. 3).

### Evaluation of the LF-RPA assay

Of the 60 oysters screened for *P. beihaiensis* infection by conventional PCR, 29 tested positive. Of these 29 oysters, 25 were LF-RPA-positive, equating to a test sensitivity of 86.2% (Table 2). Of 31 PCR-negative samples, 24 tested LF-RPA-negative, equating to a test specificity of 77.4%. In 49 of 60 test results (81.7%), LF-RPA assay results agreed with PCR results. A McNemar Chi-square test revealed LF-RPA assay results were not significantly different from those of the PCR assay (*P* = 0.549) (Table 2). Although results of LF-RPA and PCR assays were positively correlated, concordance was moderate (*K* = 0.634, i.e. *K* < 0.75) (Table 2).

**Table 2:** Performance of LF-RPA assay *versus* qPCR and conventional PCR assays for detection of *Perkinsus* spp

			PCR assay		qPCR assay	
			Positive	Negative	Positive	Negative
LF-RPA assay		Positive	25 (TP)	7 (FP)	28 (TP)	4 (FP)
		Negative	4 (FN)	24 (TN)	2 (FN)	26 (TN)
Performance characteristics	McNemar’s test		*χ*^2^ = 0.167, *P* = 0.549		*χ*^2^ = 0.364, *P* = 0.687	
	Kappa		*K* = 0.634, *P* = 0.000		*K* = 0.800, *P* = 0.000	
	Sensitivity (%)		86.2		93.3	
	Specificity (%)		77.4		86.7	
	Accuracy (%)		81.7		90.0	

*Notes*: Sensitivity = TP/(TP + FN) × 100; specificity = TN/(TN+FP) × 100; accuracy = (TP + TN)/(TP + TN + FP + FN) × 100

*Abbreviations*: LF: lateral flow; RPA: recombinase polymerase amplification; TP, true positive; FP, false positive; FN, false negative; TN, true negative

Of 30 qPCR-positive samples, 28 were LF-RPA-positive, with test sensitivity of 93.3%. Of 30 qPCR-negative samples, 26 tested LF-RPA-negative, with test specificity of 86.7%. Assuming qPCR assay results are correct, the LF-RPA assay produced correct test results 90% of the time (in 54 of 60 samples). A McNemar Chi-square test demonstrated LF-RPA assay results were not significantly different from those of the qPCR assay (*P* = 0.687) (Table 2). LF-RPA assay results were positively correlated and highly concordant with qPCR assay results (*K* = 0.800, i.e. *K* > 0.75) (Table 2).

## Discussion

We developed an RPA assay that amplifies the ITS region of *P. beihaiensis*, which, followed by rapid detection of amplicons on a lateral flow strip, enables rapid and easy visualization of test results by the naked eye. We demonstrated this assay to be more sensitive at detecting *P. beihaiensis* when combined with a lateral flow strip (LF-RPA) than when agarose gel electrophoresis (AGE-RPA), as detection limits in the former are roughly an order of magnitude lower than the latter. Improved detection arises from application of a fluorescence-labelled primer and probe in the LF-RPA assay; the reverse primer and probe were labelled by biotin and FAM, respectively, resulting in double-labelled RPA products being caught on lateral flow strips with gold-labelled anti-FAM antibodies. This fluorescence-labelled LF-RPA preparation process was simpler than immunoassays using monoclonal and polyclonal antibodies, but produced similar highly sensitive and specific results.

Although the LF-RPA assay provides results comparable to those of conventional PCR and qPCR, its advantages include: (i) a faster reaction time (less than 30 minutes for LF-RPA *versus* several hours for PCR and qPCR); (ii) convenient operation (simplified workload in LF-RPA using freeze-dried ready-to-use reagents); (iii) less energy requirement (37 °C for LF-RPA *versus* more than 55 °C for PCR and qPCR); and (iv) easier visualization of results (these can be read by the naked eye and interpreted by untrained personnel). Accordingly, the LF-RPA assay is a viable option for accurate and rapid diagnosis of perkinsosis, especially in environments where specialised equipment and trained personnel are not available.

Our *P. beihaiensis* LF-RPA assay detects plasmid DNA of *P. beihaiensis*, *P. olseni* and *P. chesapeaki*, possibly because of high sequence similarities in the ITS target region selected for primer and probe designs. Although longer primers would increase amplification specificity for *P. beihaiensis*, we could not screen these primers of 30–35 bp length [18, 24, 28]. It may be that these longer primers would lead to secondary structures and potential primer artefacts, promoting primer-primer interactions. Our shorter primers (20–25 bp) were efficient for amplification using RPA, and products were readily detected on lateral flow strips. Fortunately, although not specific to *P. beihaiensis*, these primers did not cross-react with the DNAs of other potential pathogens occurring in the culture environment. The use of shorter primers is advantageous in that primer secondary structures are avoided, and the primer-design process is simplified.

The LF-RPA assay can detect *P. beihaiensis* at levels as low as 26 copies, making it as sensitive as the qPCR assay [20, 25, 32]. This level of sensitivity theoretically renders the LF-RPA assay capable of detecting DNA from single *P. beihaiensis* zoospores, facilitating early diagnosis of *Perkinsus* infection. The LF-RPA assay appeared to be more sensitive than the conventional PCR assay. The lower sensitivity of the conventional PCR assay lead to more false negative results compared to the LF-RPA assay when detecting samples with low *P. beihaiensis* DNA copy numbers. In this study, 10% of LF-RPA assay results (6/60) disagreed with those from qPCR assays. This may be attributable to low concentrations of *P. beihaiensis* DNA in samples, as qPCR assays demonstrated most samples that produced disagreeing diagnoses had very low *P. beihaiensis* DNA copy numbers (4 qPCR-negative but LF-RPA-positive samples, 35 < Ct value < 40, data not shown). Perhaps increasing template volume, thus increasing the number of *P. beihaiensis* DNA copies, may improve problems associated with low copy numbers of *P. beihaiensis* DNA in samples [20, 24]. Further studies could also explore ways to optimize the LF-RPA assay to increase its detection sensitivity at low *Perkinsus* DNA concentrations. It should also be noted that false positive results also may be caused by contamination using LF-RPA assay, as amplification may occur when sterile gloves, tubes and pipets are cross-contaminated with target DNAs. However, we eliminated this risk by sterilizing all experimental materials, and performing all LF-RPA reactions in separate sterilized biological safety cabinets. Further optimization of the LF-RPA assay should endeavor to minimize the risk of cross-contamination, as ensuring sterilized environments in field conditions is difficult.

## Conclusions

We developed a novel LF-RPA assay for detection of *Perkinsus* infection in molluscs that is relatively simple to use, provides accurate results and enables rapid diagnoses. While our intention had been to establish a *P. beihaiensis*-specific LF-RPA assay, the one we do develop detects multiple *Perkinsus* taxa. The short PCR primers we develop for this LF-RPA assay simplify the primer-design process. Our novel LF-RPA assay has a fast reaction time (less than 30 minutes) and can detect the DNA of multiple *Perkinsus* species as low as 26 copies. Future research should focus on developing simple DNA extraction methods that do not require professional laboratories and complicated extraction procedures, to facilitate application of this LF-RPA assay in the field.

## Data Availability

All data generated or analyzed during this study are included in the article.

## Abbreviations

AbHV: abalone herpes-like virus
AGE: agarose gel electrophoresis
CT: threshold cycle
FAM: carboxyfluorescein
FN: false negative
FP: false positive
ISH: *in situ* hybridization assay
ITS: internal transcriber spacer
LAMP: loop-mediated isothermal amplification
LF: lateral flow
OIE: World Organization for Animal Health
OsHV-1: ostreid herpesvirus 1
PCR: polymerase chain reaction
qPCR: quantitative polymerase chain reaction
RFTM: Ray’s fluid thioglycollate medium
RPA: recombinase polymerase amplification
THF: tetrahydrofuran
TN: true negative
TP: true positive
